# Symbolic access: medical students’ awareness of institutional culture and its influence on learning, a phenomenographic study

**DOI:** 10.1186/s12909-023-05001-w

**Published:** 2024-01-04

**Authors:** Dina-Ruth Lulua, Shirra Moch

**Affiliations:** 1 Health Science Education and Social Accountability, Faculty of Health Sciences, University of Cape Town, Cape Town, South Africa; 2 Centre for Health Science Education, University of Witwatersrand, Johannesburg, South Africa

**Keywords:** Access, Symbolic, Undergraduate student, Institutional culture, Phenomenography, Learning, Medical community

## Abstract

**Background:**

The discussion of access in medical education has its focus largely on physical and epistemological access, leaving a qualitative gap regarding sociocultural factors which enable access in this context. This study introduces and defines symbolic access, a concept with a specific lens on sociocultural inclusion, and the influence it has on student learning within the South African medical education landscape.

**Methods:**

A phenomenographic design was used to explore students’ conceptions of symbolic access and its impact on learning. One-on-one exploratory interviews were conducted with fifteen final year medical students at the University of Witwatersrand in Johannesburg. Interviews were analysed using Sjöström and Dahlgren‘s seven-step phenomenography model.

**Results:**

Four categories of description were induced, which described students’ understanding of symbolic access, these were rejection, disregard, invalidation, and actualization. Four dimensions of variation were discovered expressing the diversity of events which informed the collectives’ understanding of the phenomenon. These dimensions were; interactions with educators, peer relationships, educational environment, and race. Categories of description and dimensions of variation formed the Outcome Space, a visual representation of the student experience of symbolic access. The outcome space had a double narrative related to symbolic access; exclusion (major) and actualization (minor). Medical student’s chief experience within the medical community was exclusion, however clinical immersion, meaningful participation, peer-relationships, and clinical skills lessons facilitated community enculturation, and impacted learning.

**Conclusion:**

Despite deeply exclusionary experiences throughout their programme, medical students articulated a paradox of both awareness and no awareness of symbolic access. The awareness of symbolic access was predominantly influenced by clinical experiences and clinical immersion during the pre-clinical and clinical years of study. Further, descriptions of valuable learning experiences were connected to clinical events and the involvement with patient care. This study suggests that the actualization of symbolic access and description of meaningful learning experiences are linked. Medical educationalists should design undergraduate curricula with early clinical immersion at the fore and explore symbolic concepts pertaining to access, as they are linked to transformative learning experiences for the medical student.

## Introduction

Over the past fifty years there has been an intentional widening of access in higher education, this expansion focused primarily on physical and epistemological access with limited attention on access through the symbolic lens [1, 2]. The symbolic lens of higher education constitutes its culture, traditions and implicit communal codes which shape an institution as a community. This symbolic space is bound by intangible yet present borders that define some people and groups as included while excluding others [3]. The discussion of access in higher education, therefore, goes beyond formal admission into physical spaces or the availability of certain knowledge, to include the symbolic entry into the community or institution; that is symbolic access.

**Fig. 1:**
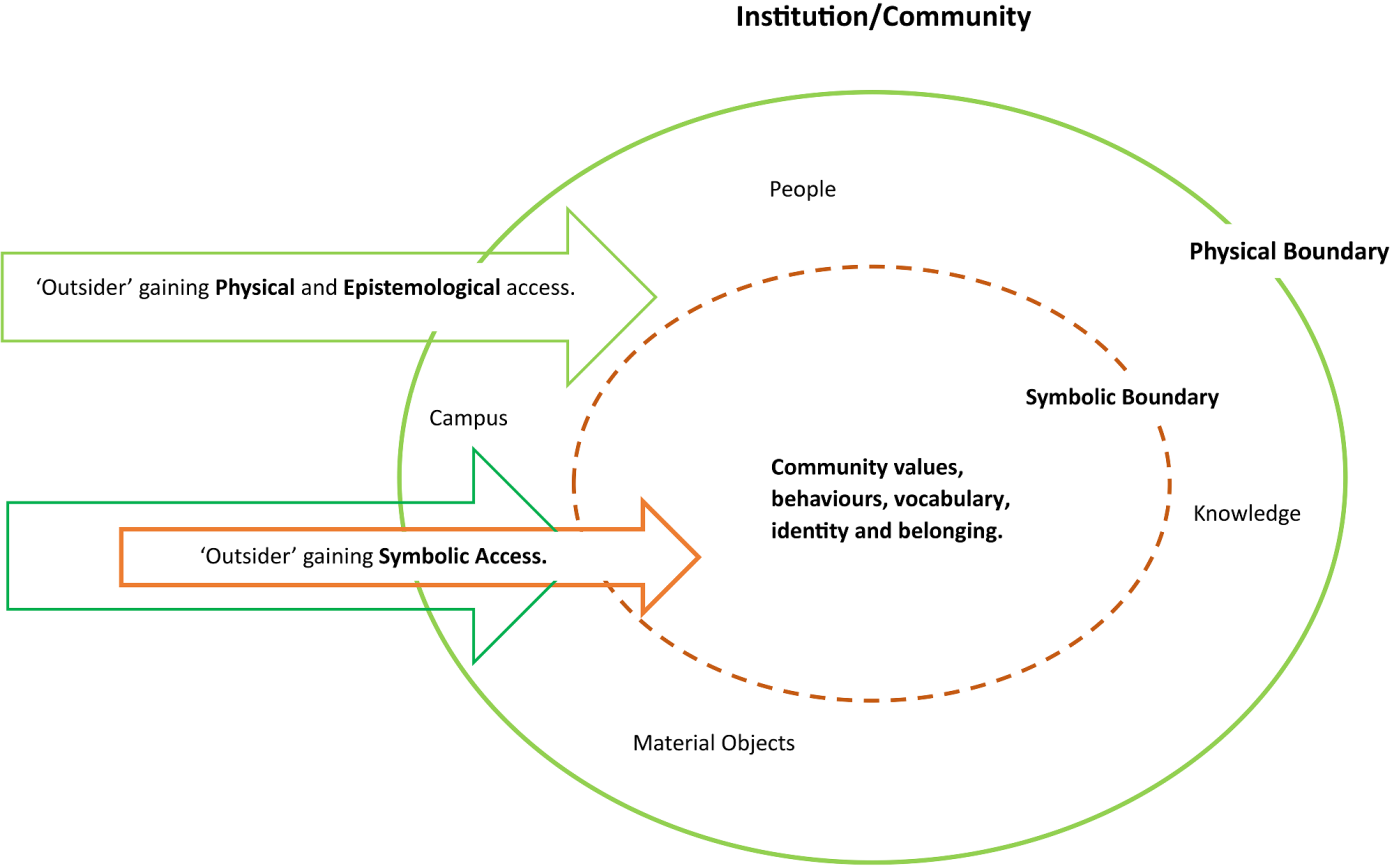
Visual representation of symbolic access

Symbolic access is an emerging concept; as such the authors propose a current working definition as *‘an ongoing set of experiences, feelings and events which facilitate an ‘outsiders; enculturation and identity formation within a community’* (Fig. 1) [4–6]. Symbolic access is facilitated within a community through various activities including, but not limited to; curiosity, relationship development, socialization, and intentionality [4, 7, 8].

## Medical education, symbolic access, and situated learning theory

The medical community has deep rooted cultures, and students admitted into medicine come eager to learn what is necessary to be part of the community, fostering hopes of developing their identity as a junior doctor. Over the years the medical community has been encouraged to not only supply students with programme admission and course-based content; but to be cognisant that through professional relationships and socialization they facilitate symbolic access for the medical student [9].

Lave and Wenger’s [7, 8] Situated Learning Theory asserts that learning is a social process, shaped by the development of relationships and socialization between newcomers (students) and old timers (established community members) in a Community of Practice (CoP).

They conclude that learning has happened when a newcomer changes their participation within the community. Symbolic access and learning intersect at the point of relationship development and socialization within a community, the study of symbolic access in higher education therefore is also an exploration of factors that influence student learning in higher education.

### Literature review

Considerable educational research is driven by the question: “What factors influence student learning in higher education?” [10–13]. Commonly documented influences include student learning approaches, teaching strategies, demographic background, and the learning environment [14–18]. While these factors have been explored extensively there is need to focus on sociocultural factors as they relate to student learning. Sociocultural factors include the relationships, culture, traditions and values of a community or institution [19]. Leibowitz et al. [20] explain that in order for learning to successfully occur, continual consideration and reflection needs to be placed on the social, cultural, and community factors that promote or constrain learning. Therefore, an inquiry into how students recognize, and access institutional culture is warranted.

### Symbolic access and the hidden curriculum

Educator-student socialization, relationship development and enculturation largely occur in the hidden curriculum, an informal yet influential curriculum in medical education [21]. The process of symbolic access is situated therefore within the hidden curriculum. Over the last two decades medical education has produced growing literature on the hidden curriculum, however, Bandini et al. [22] highlight that there has been a lack of specificity in this global research. Asserting that there are multiple ways the hidden curriculum presents itself; it is not just a blanketed singular curriculum. Lawrence et al. [23] encourage researchers to make explicit the details and nuances of their hidden curriculum, as being detailed will allow policymakers and curriculum developers to better identify literature related to their own needs and initiatives. The exploration of symbolic access in medical education constitutes a nuance of the hidden curriculum.

Two thirds of current literature on the hidden curriculum is limited to North America. As a result, the production of more contextualized studies from areas such as Africa has been strongly encouraged to adequately represent hidden curricula in the African medical school context [23]. The study of symbolic access within a South African context yields contextualized perspectives on the hidden curriculum.

### South African higher education and symbolic access

The South African higher education setting is uniquely framed for the exploration of symbolic access. South Africa has a devastating socio-political history, which created distinctly separatist higher education settings and cultures under the apartheid regime [24]. A distinguishing feature of the apartheid higher education regime was the unequal access to academic, structural, cultural, and social resources in the education of different racial groups. As a result of these disparities, one of the main priorities of the 1994 democratically elected government was to redress the inequalities within the higher education sector, by adopting policies that would widen access to higher education for all South Africans [25, 26].

While significant achievements regarding access policies were realized, the higher education mandate of the new government focused primarily on physical and epistemological access in higher education, at the expense of issues such as institutional culture and symbolic access [26–29]. The journey of how students would adopt institutional culture was not deeply considered. Entry into higher education did not necessarily equate to student learning success, as this physical provision did not address inclusion beyond university admission [29–31].

Twenty years into democracy, the South African government was confronted with this symbolic oversight, in what Jansen et al. [32] describe as one of the most intense and violent student protests in higher education, the #RhodesMustFall and #FeesMustFall movements. Two factors attributed to the 2015–2016 country-wide student protests: the alienating cultures of historically white universities, known as #RhodesMustFall; and the discriminatory cost of higher education known as #FeesMustFall. The #RhodesMustFall movement brought to light the ills of cultural exclusion, experienced post 1994 by the black scholar and black academic [33].

Symbolic access to institutional culture appears to be a persistent stumbling block in the South African higher education space. If left unexplored new health science students and new generations of future health care workers will be forced to exist on the periphery of a community, they should be a part of. It is increasingly important for educators in higher education to be cognisant of institutional culture and the phenomena pertaining to it, as it empowers educators to thoughtfully engage students and take responsibility for the role, they play in student learning [34].

The South African medical students’ experience of access to institutional culture and the impact it has on learning has not been formally researched or reported within the context. Given the nation’s past pain, present awakening, and future aspirations there is need to investigate this space further. This body of research aimed to investigate the impact of institutional culture on medical student learning, by asking the question, ‘how does symbolic access to institutional culture affect learning for the medical student?’. To answer this question researchers explored students’ awareness of symbolic access in the medical community, and further what influence symbolic access had on their learning.

### Methodology and methods

This study was rooted in an interpretivist paradigm and used phenomenography, a growing qualitative methodology within health sciences [35, 36]. Phenomenography aims to answer specific questions about thinking and learning and helps describe the variation of ways that people understand the same phenomenon [37, 38].

### Study setting

A unit for undergraduate medical education at a research-intensive university in Johannesburg, South Africa was chosen as the study setting. It is important that the setting provides the researcher access to study participants who can give insight and relevant information on the topic being studied and for the researcher to elaborate on the social context of the study setting, as it adds insight to the investigation [39]. The medical school, where this study was conducted, is a historically White Anglo-Saxon influenced university, which has its roots in exclusion and segregation based on race, gender, class, and religion. Over the years there has been intentional diversification of the medical school community in this institution; which now consists of a population closely representative of the South African population at large.

At the selected university the medical degree (MBBCh) is 6-years long. There are two entry points into the programme: as a secondary school leaver into the first year of the programme or as a graduate into the third year as part of a Graduate Entry Medical Programme (GEMP). First to fourth year students spend 90% of their almanac in didactic teaching, while fifth- and sixth-year students spend 95% of their academic calendar in the clinical setting as they rotate between three academic teaching hospitals in Johannesburg and Soweto.

### Study participants and sampling

Participant selection for phenomenographic studies requires the researchers to make strategic and purposeful efforts to maximise diversity of participants selected, this ensures inclusive representation of participant experiences [40]. The entire final year medical class was invited to participate in the study. The researchers had no teaching or examination responsibility with this cohort of students, and no incentive was provided for participation. This study was conducted in 2020, and due to COVID-19 pandemic academic disruption, and lockdown regulations convenience sampling, instead of purposeful sampling, was conducted. Participants were selected based on their availability of time, access, and willingness, resulting in a realized sample. The realized sample mirrored the study population in key areas (Table 1) and was therefore sufficient to provide insight into the study population. The sample size of this study was 15 participants, phenomenographic studies typically suggest a sample of 10–15 participants as sufficient to capture variation and reach saturation [40]. There is no recommended upper limit currently, however sampling should ensure that the resulting amount of data remains manageable [41, 42].

Following an invitation email sent to the entire class, 19 final year students showed interest in being participants, the first 15 participants who were readily available were interviewed and became the realized sample.

**Table 1:** Study population and realized sample demographics

Realized Sample (n = 15)			Study Population (n = 321)
**Race**	African	N = 8 (53%)	N = 122 (38%)
	Asian	N/A	N = 4 (1.2%)
	Coloured	N = 1 (6.7%)	N = 15 (4.7%)
	Indian	N = 1 (6.7%)	N = 69 (21.5%)
	White	N = 5 (33.3%)	N = 111 (34.6%)
**Age Range**	20–22 years	N/A	N = 13 (4.1%)
	23–24 years	N = 10 (66.7%)	N = 201 (62.6%)
	25–26 years	N = 2 (13.3%)	N = 51 (15.9%)
	27–28 years	N = 2 (13.3%)	N = 46 (14.3%)
	> 30 years	N = 1 (6.7%)	N = 10 (3.1%)
**Academic Performance**	Top Performing	N = 4 (26.7%)	N = 53 (16.5%)
	Middle Performing	N = 9 (60%)	N = 156 (48.6%)
	Lower Performing	N = 2 (13.3%)	N = 112 (34.9%)
**Gender**	Female	N = 9 (60%)	N = 179 (60.7%)
	Male	N = 6 (40%)	N = 126 (39.3%)
**Entry to Medicine**	School Leaver	N = 12 (80%)	N = 204 (63.6%)
	Graduate	N = 3 (20%)	N = 117 (36.4%)
**Student Country of Origin**	Local (South African)	N = 12 (80%)	N = 309 (96.3%)
	International	N = 3 (20%)	N = 12 (3.7%)

### Positionality of the researcher

As the primary researcher I [DRL] recognize and acknowledge my fixed and subjective positionality that may have influenced this research study. I am a 35-year-old female of African heritage. I am a graduate of the MBBCh programme that was under investigation in this study. I have my own 6-year lived experience of institutional culture within the institution as a young Black female student. I draw on racial, social, educational, symbolic, theological, scientific and creativity theories to make sense of the world and am constantly aware of my race, gender, and faith as I navigate every day. I acknowledge that my positionality shapes my work and influences my interpretation, understanding, and belief in the truthfulness and validity of the participant experiences.

### Data collection

All interviews were conducted as one-on-one in-depth interviews, in keeping with phenomenographic guidance [43, 44]. Due to the COVID regulations all interviews were held online via the Microsoft Teams (MS Teams) application, which was freely available to students in the university. DRL conducted all interviews, which ranged from 30 to 60 min in length. Data was collected from April to September of 2020. Two exploratory questions were created by the researchers these were; (1) Please tell me about your experience of learning at Wits Medical School? And (2) Can you elaborate on what impacted your learning? And two prompt questions were formulated, to solicit further depth from participants, these were 1)What is your experience of educators/doctors at medical school? And 2)What is your experience of the medical school environment. All four questions were assessed and revised in collaboration by both researchers prior to data collection, additionally the research question, aims and objectives were referred to when creating the interview questions in order to make sure they would satisfy the study. During and after each interview DRL took notes of how best to conduct future interviews, discussing previous interviews with SM. DRL utilized the notes in subsequent interviews. As per phenomenographic methodology all 15 interviews were recorded and transcribed verbatim for subsequent analysis [40].

### Data analysis

**Fig. 2:**
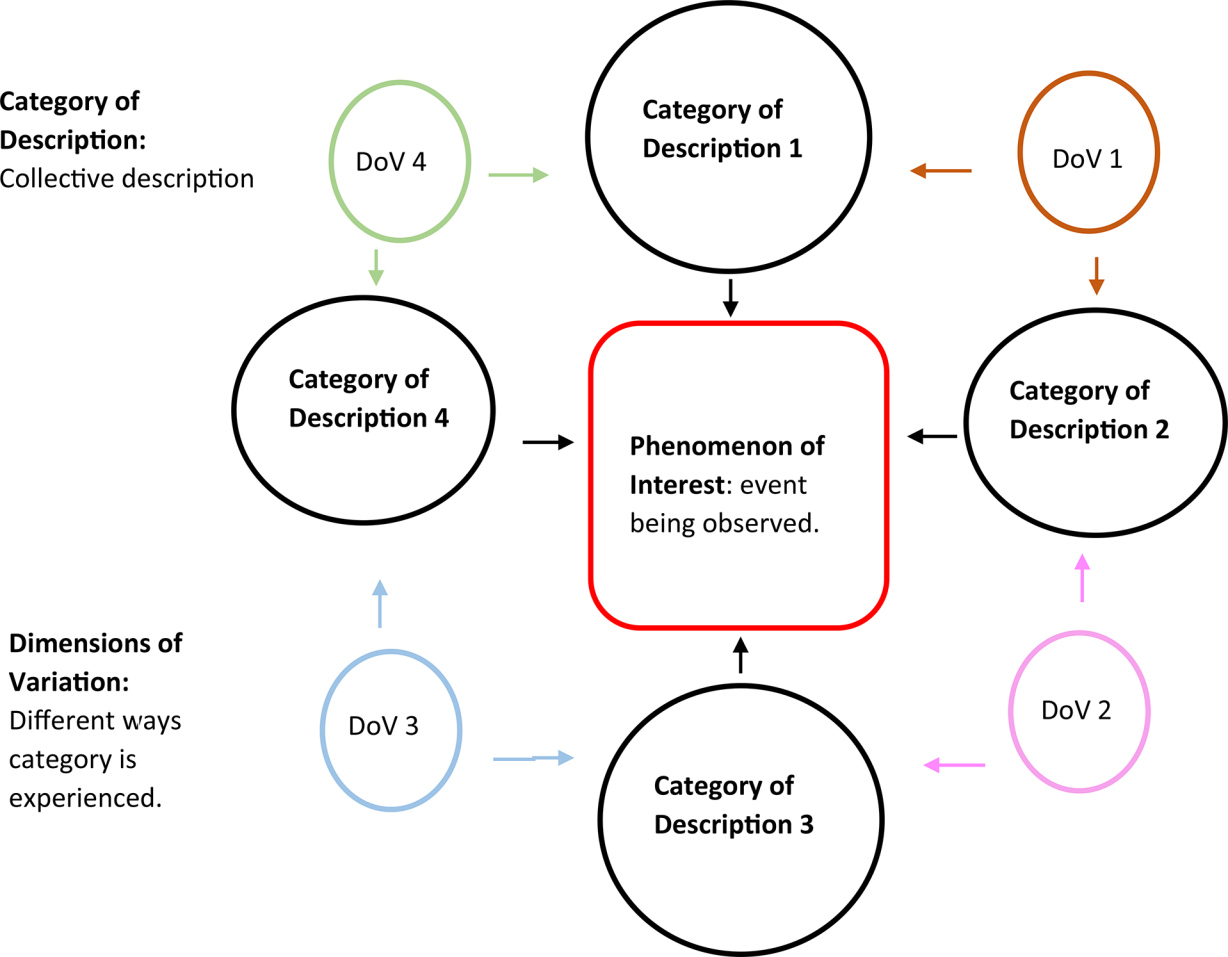
Interaction between categories of description and dimensions of variation

Interviews were analysed using Sjöström and Dahlgren‘s seven-steps for phenomenographic data analysis [45]; this method was chosen as it is easy to understand for the novice researcher and did not conflict with the other available phenomenographic processes [46, 47]. Steps in the data analysis process is depicted in Table 2. The aim of the phenomenographic analysis is to produce an Outcome Space, this is a visual representation of the way the collective understood the phenomenon. The outcome space is populated by two elements, namely Categories of Description, which represent the qualitatively different ways which participants understood the phenomenon and the Dimensions of Variation, which represents the events that facilitated those understandings [48, 49]. The first step of phenomenographic analysis result in the development of Categories of Description; continued analysis reveals the Dimensions of Variation (Fig. 2).

**Table 2:** Steps in data analysis Sjöström and Dahlgren [48]

Sjöström and Dahlgren‘s Seven-steps	DRL Process of Analysis
Step 1 Familiarization	The researcher printed and read all transcripts repeatedly while simultaneously listening to the audio recordings, in order to familiarize herself with the data. Notes and sections of each interview were manually highlighted. The researcher immersed herself in the data to ensure in-depth understanding of the text and to get accuracy of context.
Step 2 Condensation	Relevant recurring feelings, words, and experiences participants used were identified, grouped together, and recorded as patterns. This was done in order to uncover repeated patterns or similarities in the data.
Step 3 Comparison	Researcher studied and discussed patterns with supervisors in order to identify similarities and differences in the patterns.
Step 4 Grouping	Patterns were sorted into initial Categories of Description according to the similarities of their essence and how the categories described the participant’s experience of the phenomenon - symbolic access.
Step 5 Articulating	Categories were read and discussed between researcher and supervisors to identify the essence of each category. The main aim of this step is to set up boundaries among the categories.
Step 6 Labelling	After confirming the categories, the next step was to distinguish features between them, these become the Dimensions of Variation, and label them accordingly.
Step 7 Contrasting	Discussion and reviewing of categories of descriptions were held between the researcher and supervisor. Categories were contrasted and thoroughly defined in order to be clearly distinguished and arrive at the four categories in this study. The process of articulation, labelling and contrasting (step 5–7) was iterative, which is true to the nature of phenomenographic data analysis. The end goal was to define categories which best represented the qualitative variations of the phenomenon from the participants’ responses.

All interview recordings were printed and manually analysed by the principal researcher (DRL) who induced categories and variations. After initial development the principal researcher discussed categories, variations, and findings with the senior researcher (SM) during weekly meetings over the course of one year. The final categories and variations were established through an iterative process between both researchers. This collaborative discussion is fundamental in the phenomenographic data analysis process as it helps to avoid researcher bias and preconceived notions [50].

## Results

Four categories of description were induced, Rejection, Disregard, Alienation and Actualization.

These categories expressed the collectives experience of the phenomenon. In phenomenography it is impossible for one quotation to illustrate all aspects of the category described, but one appropriate quote can be selected which emphasizes the differences between categories, more than their commonalities [51]. Below is a brief description of each category followed by relevant quotations.

### Category of description 1: Rejection

Rejection was the experience of the collective feeling unwanted in the community. It was most prominent during the pre-clinical years and carried into the clinical years in varying intensities.

In the earlier years, you really don’t have a relationship with most of your teachers or your educators, you’re on main campus and you’re thinking, am I even studying Medicine (Participant SBG13).

The one doctor said she never signed an agreement to teach students. And she said that blatantly in our face, and we’re like, were you never once a student? (Participant GECG15).

### Category of description 2: Disregard

Disregard was the feeling of not being recognized by the community. It was experienced due to various events, from poor teaching efforts during the pre-clinical years to the lack of acknowledgement from clinical educators during the clinical years.

Many times, a consultant would come do the ward round with everyone there, no hello to us or anything, then once the round is over, they leave, without even recognizing we [students] are there. And that’s it (Participant SBB14).

To me it seemed like it was an additional duty of the clinician to deliver a lecture, but not necessarily a primary duty, or seen as a primary thing to be accomplished– or an important thing to be accomplished. More as a side thing (Participant SWB8).

### Category of description 3: Alienation

Alienation was the experience of separation and isolation from the community. Although it was experienced by the general collective, it was most pronounced for the Students of Colour (SoC)*^1^ collective.

### General alienation

“For me to be honest, Medical School is just not welcoming or friendly. During the pre-clinical years, you’re basically sitting in lecture theatres all day, then the clinical years are two years of feeling unwanted” (Participant SWG1).

### Racial alienation

Some doctors would just come; they don’t say anything to black students. So, whenever they ask questions, [during ward rounds] they ask the white students. In my experience some of them focused more on the white students.

### SBB14

… I noticed that you really get treated differently in terms of race. Like, on our first day [in the ward]. We are a group of three brown students– so we get there, and there was no introduction whatsoever. It was just like, oh hi, you’re the students, okay, let me show you where the stuff for bloods is…The following week three students joined us and both of them were Caucasian. And the way they were greeted; there were like formal introductions and at each patient there was a short summary, or kind of an assessment thing. And the whole week prior to that, we had not ever been acknowledged as being in the ward.

### GECG15

I don’t know, but like a lot of Black people fail. There is a lot of Black people failing [compared to] white people. I don’t think it’s because white students are smarter than the black students. Like, somehow the environment makes it easier for them to work.

### SBB7

“There weren’t many black doctors, actually. Within each block, maybe out of 20 tuts, four would be from black doctors. Obviously, it’s different [when you have a Black senior]. Black doctors were usually nice.

### SBG13

#### Category of description 4: Actualization

This category expressed the collectives’ realization of symbolic access; three broad events contributed to this realization: (1) Clinical immersion and meaningful participation during the clinical years (2) Relationships with peers, and (3) Clinical skills teachings during the pre-clinical years (3rd and 4th year).

#### Clinical immersion

It was invaluable being in the hospitals, to actually manage the patients, see the conditions that you’ve heard the theory about (Participant SBB7).

#### Meaningful participation

“And then I did student internship. I thought that was really cool… I think the nice part of that was that for once there wasn’t such a focus on bedside tutorials and I was working with the team” (Participant SBB4).

#### Peer relationships

… When you go to Med School, you have to rely on your friends, I would say, to make the environment more lively and do well [academically] (Participant SIB2).

#### Clinical skills lessons

And then, in 3rd and 4th year with clinical skills, I felt like the teaching was good and educators were very nice people. Very nice. They understood students, what students need, you know. So, I think clinical skills in 3rd and 4th year, we had good educators (Participant SBG4).

#### Dimensions of variation

In phenomenography categories of description are experienced through dimensions of variation, dimensions are a collective of similar events found in the data, through which categories are experienced, dimensions of variation are not supported by quotes rather they represent an event which informed the category. The four dimensions of variation that were discovered were: (1) Interactions with Educators, (2) Peer Relationships, (3) The Educational Environment and (4) Race.

#### The outcome space

**Fig. 3:**
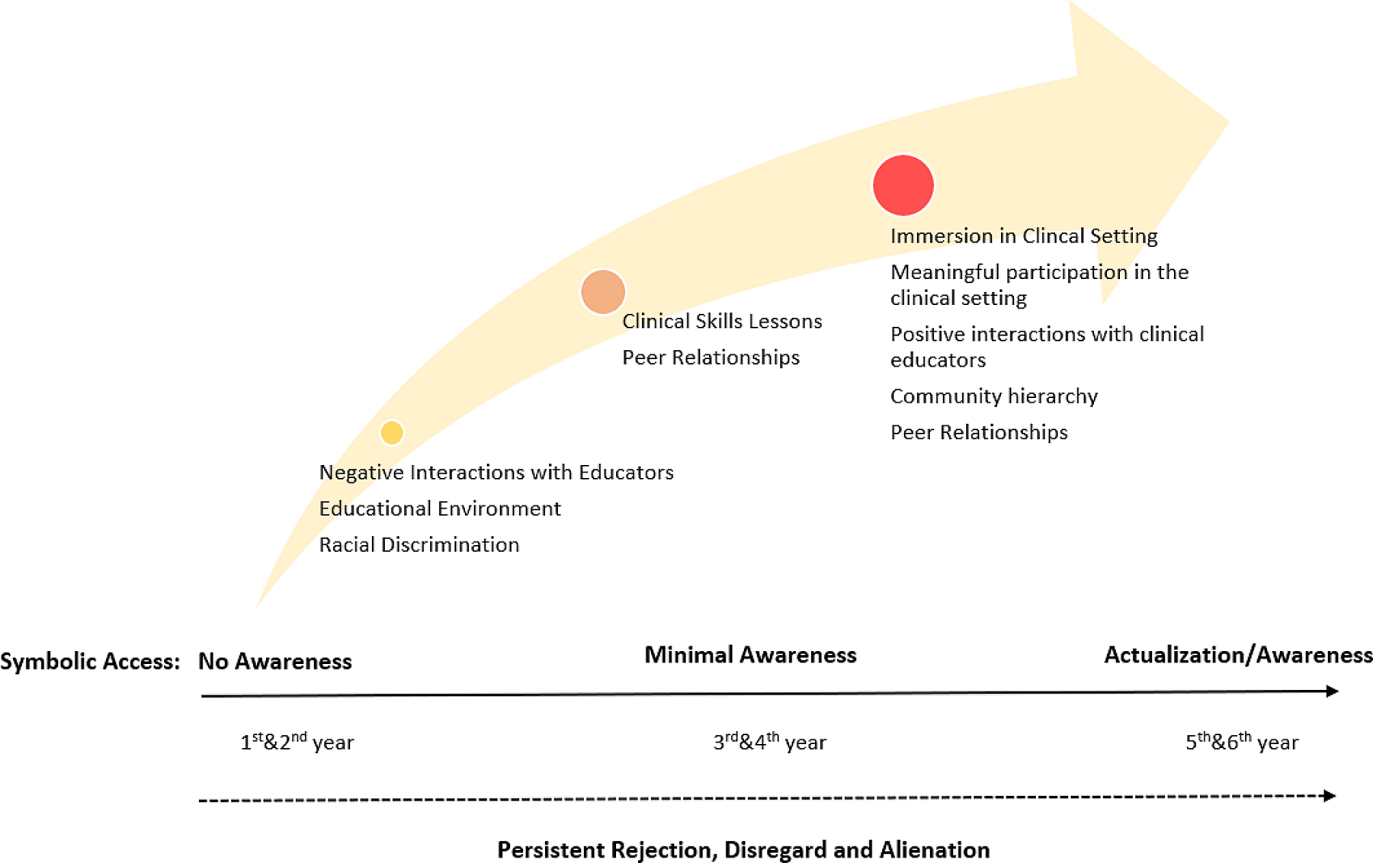
Chronological outcome space

The Outcome Space is the integration of the categories and dimensions, it gives a visual representation of how the phenomenon was experienced at a collective level [49, 52]. It can be represented as a diagram, table, or figure. Three outcome space types exist: hierarchal inclusive, with categories presented in taxonomy format, climatic, in which categories are arranged according to the level of the explanatory power, and chronological, which represents the development of the participants’ experience of the phenomenon [48]. This study presents its outcome space in two ways, first using a chronological figure (Fig. 3), this shows the evolution of how participants experienced symbolic access throughout the programme. The second outcome space representation is in table format (Table 3), it is a more detailed representation of the outcome space. A discussion of the outcome space will be elaborated in the discussion section.

## Discussion

This discussion is divided into three parts; **Part I**: The Outcome Space: what the data means, **Part II**: Students’ awareness of Symbolic Access: Do you feel like you’re part of the team? and **Part III**: Does symbolic access impact learning? These separate sections discuss what was found in the outcome space, linking it to the research questions, aims, objectives and the literature.

### Part I

The Outcome Space, what the data means.

Two experience narratives were described in the outcome space, the first major narrative was of exclusion, which was largely influenced by racial discrimination, negative interactions with educators and the educational environment. These narratives are elaborated in the following paragraphs.

### Exclusion

Exclusion was a common narrative described by the collective, of particular note was the SoC collectives experience of exclusion. The SoC spoke of alienation on two levels, the general alienation of being a student in the medical community and specific alienation linked to being a SoC in the medical community. While the general collective described being ignored during ward round, the SoC collective further emphasized that when recognition did occur it was directed to the White students. SoC further explained seeing White students acknowledged in the community in ways that they were not. During one occasion White students received a welcome and orientation to the ward which was not done for the SoC group who joined the clinical unit the week before. The SoC collective described having to work twice as hard academically to prove themselves in environments not created for them to succeed, with few role models to ‘lean into’ for mentorship and support. Unfortunately, narratives of racial exclusion within the South African medical community are not unique and have been retold by various generations over the years. During the colonization and apartheid, the medical community physically alienated SoC from academics, spaces, and patients. Over the years, as physical and epistemological inclusion have been granted, symbolic alienation persists[53].

During the first four years of the programme the collective described a sense of rejection and illegitimacy, based on their physically separate location from the community and interactions with educators in which educators would express that teaching was not their priority. Although students had the title of medical student, it did not translate to their lived experience and instead of developing community identity, the collective identified as general university students. In the clinical years student interactions with clinical educators was erratic and inconsistent. Experiences of exclusion were interspersed with some recognition and inclusion. The collective described little acknowledgement from seniors, and feelings of not being a priority, especially to those designated to deliver bedside tutorials. South Africa, similar to other low to middle income countries, faces enormous healthcare challenges. The extent of the burden of disease, coupled with the lack of adequate health workers to provide services exacerbates the situation[54]. As a result of this strain senior clinicians juggle between delivering adequate service delivery and quality student teaching. Poor acknowledgement, little student interaction and strained teaching is not solely a reflection of the community but of structural factors which impact the community.

### Inclusion

The second minor narrative from the outcome space was of inclusion and the collective’s awareness of symbolic access, which this study defines as actualization. Clinical immersion and meaningful clinical participation were significant actualization events for the collective. Being in the hospital, entrusted to manage patients, and the acknowledgement from some senior clinicians boosted the collective’s confidence and sense of legitimacy within the community. Actualization due to clinical immersion was particularly striking for the SoC collective who, despite deeply exclusionary experiences, described an awareness of belonging, due to clinical work and patient interaction.

Peer-peer relationships between junior and senior students was the first ‘welcome’ the collective received into the community. These informal relationships were a source of comradery, identity affirmation and aspiration. Senior students shared strategies of how to become successful both academically and socially in the community. Clinical skills teaching sessions were profoundly inclusionary experiences for the students. During these teaching lessons the collective described positive community socialization with their clinical educators, and important participation in community traditions like wearing scrubs, using medical equipment, and performing clinical examinations. Clinical skills sessions were the early foundation for symbolic access.

**Part II** Students’ awareness of Symbolic Access: Do you feel like you’re part of the team?

The collective described the paradoxical experience of both an awareness of and no awareness of symbolic access within the medical community. This paradox was highlighted by the collective’s explicit sense of community exclusion coupled with their ability to self-locate themselves within the medical community.

The main factors which contributed towards the collective’s awareness of symbolic access were found to be; being in the hospital, participating in bedside learning opportunities, managing patients, performing clinical skills, working with future colleagues and peer relationships. Due to the heavy patient load of the South African academic healthcare setting, the undergraduate medical student has rapid engagement with patient care and management during their clinical years. Students are often entrusted to manage a variety of patients, from admission to discharge, under the supervision of an immediate colleague e.g., intern, medical officer, or junior registrar (resident). Medical students move from experiences of minimal involvement with patients during their pre-clinical years, to daily intense patient interaction in the clinical years. This rapid clinical immersion contributes greatly to the collective’s awareness of symbolic access, despite early exclusionary experiences.

#### Part III

Does symbolic access impact learning?

During the pre-clinical years teaching was didactically delivered to classes of 300 + students, this meant poor educator-student interactions for the better part of four years and learning described as isolated and disconnected from ‘real medicine’. One exception to the negative pre-clinical learning experience was clinical skills sessions. These teaching and learning sessions were valued by the collective, who appreciated small group learning, and described the learning environment as supportive and conducive.

Similarly learning experiences during the clinical years were largely positive. Bedside teaching in smaller clinical groups with real life patients and experiential learning bridged theory-practice gaps and created meaningful learning experiences for the students. Clinical learning is said to be the cornerstone of medical education, as it is in this learning environment that students are able to participate in the medical community and develop both their clinical skills and professional identity. Medical students often perceive the tangible difference between the pre-clinical and clinical curricula; and are able to articulate these changes in their learning due to the vast contrast of experiences [55].

Events which resulted in students’ actualization of symbolic access i.e., clinical skills teaching, clinical immersion and participation also resulted in students’ perceptions of meaningful learning experiences. As the collective gained symbolic access their learning ‘came to life’ and shift from disconnected, isolated, and dead-end learning to applicable, contextual, and valuable.

## Conclusion

The South African higher education context is historically located in a culture of segregation and discrimination, the concept of symbolic access is vital if we, within this context, want to truly examine issues of institutional culture and how it impacts learning. This study suggests that symbolic access functions as a ‘key’ that unlocks the process of community enculturation and identity development, and further that it facilitates positive learning experiences for the medical student. In medical education symbolic access as well as meaningful learning was facilitated through clinical events.

### Implications for education and future research

Informed by this study, the researchers propose the following recommendations to those within the South African medical education community who are responsible for curriculum design: The implementation of learning events and opportunities which promote educator-student interaction and support, this may include small learning groups, increased lecturer accessibility and formalizing near-peer engagements. Additionally, the researchers recommend the inclusion of early clinical engagements (commencing in the first year of the programme) and sustained clinical exposure for the mid to senior medical student. Early engagements would kickstart community enculturation and professional identity formation. Sustained clinical exposure would serve to facilitate symbolic access, even across deeply exclusionary barriers.

Importantly South African medical educationalist need to engage students of colour in regular discussion forums and workshops with the specific intent of learning about and acknowledging the student’s lived experience of the community. From these engagements educators should consider ways to change and co-create an inclusive medical school curriculum, culture, and environment. Until students of colour see themselves represented in the curriculum and community they will continually exist on its periphery.

Further scholarship of the concept of symbolic access, both at a local and global level, is recommended. This research should focus on the development of its definition, interrogation into the ways in which symbolic access can be used as a tool to make institutional culture accessible to ‘outsiders’ and the role of symbolic access in higher education for students and staff who belong to marginalized groups.

### Study limitations

This study was conducted in one institution, making the findings contextual, limiting generalizability. There was scarcity of research on the phenomenon under investigation, symbolic access, meaning there was minimal literature to support or elaborate on the phenomenon. The study methodology chosen was labour and time intensive and the sample size may have had more variation of experiences if it was larger, although it was within acceptable limits for a phenomenographic study. The involvement of researchers meant that data could not be completely without the bias of the researchers, who developed the categories of description. Further the interpretation of participants words, which resulted in the categories of description, was influenced by the philosophies and interests of the researchers. However only interpretations supported by the data were taken. Readers and researchers are therefore open to analyse the categories based on the data given and arrive at a different interpretation [49].

**Table 3:** Tabular outcome space

OUTCOME SPACE				
Categories of Description	Dimensions of Variation			
	Interactions with Educators	Relationship with Peers	Educational Environment	Race
**Rejection**	**No initiation** by educators to develop educator-student relationships.	No rejection described in relation to relationship with peers.	1st -4th year: Students **not welcomed** by medical community. 5th &6th year: Feelings of **illegitimacy** in the community by students.	**Exclusion** and **poor community identification** by SoC.
**Disregarded**	1st -4th year: Students **not recognized** by community. Clinical Years: Students **ignored** during significant community events.	No disregard described in relation to relationship with peers.	1st year: Students **completely ignored** by medical community. 2nd -4th year: Students on medical campus but **not prioritized**. 5th &6th year: stressful often with **little support** from educators.	**Neglect** and **othering** of SoC based on race.
**Alienation**	Significant **isolation** during early years of the programme.	Minimal academic alienation, based on race, described by collective.	1st − 4th year: Students **separated** from the medical community in lecture theatres.	Sense of **estrangement** from the community.
**Actualization**	3rd and 4th year: Clinical Skills educators source of community **acceptance.** 5th &6th year: **Recognition** and **prioritization** close to graduation.	Facilitated a sense of **community** and **identity development**. Students **‘seen’** by peers. **Source of comfort** and **guide** for students to navigate the academic and clinical space.	3rd and 4th year: Clinical Skills lessons opportunity to learn skills to be used in future. **Safe environment.** 5th &6th year: Opportunity to **meaningfully participate**.	Positive experiences of **encouragement** by seniors of same race.

## Data Availability

The datasets used and/or analysed during the current study are available from the corresponding author on reasonable request.

## Footnotes

1 The terms Students of Colour (SoC) and People of Colour (PoC) are used to connote all those groups that were disenfranchised by apartheid and is thus inclusive of Black African, Coloured (mixed race) and Indian groups.

## List of Abbreviations

MBBCh: Bachelor of Medicine and Bachelor of Surgery Degree
CoP: Community of Practice
MS Teams: Microsoft Teams
SoC: Students of Colour (African, Coloured, Indian)
Wits: University of the Witwatersrand
